# Microbial production of short chain diols

**DOI:** 10.1186/s12934-014-0165-5

**Published:** 2014-12-10

**Authors:** Yudong Jiang, Wei Liu, Huibin Zou, Tao Cheng, Ning Tian, Mo Xian

**Affiliations:** CAS Key Laboratory of Bio-based Materials, Qingdao Institute of Bioenergy and Bioprocess Technology, Chinese Academy of Sciences, No. 189 Songling Road, Qingdao, 266101 China; University of Chinese Academy of Sciences, Beijing, China

**Keywords:** Propanediol, Butanediol, Microbial synthesis, Downstream technologies

## Abstract

Short chain diols (propanediols, butanediols, pentanediols) have been widely used in bulk and fine chemical industries as fuels, solvents, polymer monomers and pharmaceutical precursors. The chemical production of short chain diols from fossil resources has been developed and optimized for decades. Consideration of the exhausting fossil resources and the increasing environment issues, the bio-based process to produce short chain diols is attracting interests. Currently, a variety of biotechnologies have been developed for the microbial production of the short chain diols from renewable feed-stocks. In order to efficiently produce bio-diols, the techniques like metabolically engineering the production strains, optimization of the fermentation processes, and integration of a reasonable downstream recovery processes have been thoroughly investigated. In this review, we summarized the recent development in the whole process of bio-diols production including substrate, microorganism, metabolic pathway, fermentation process and downstream process.

**Electronic supplementary material:**

The online version of this article (doi:10.1186/s12934-014-0165-5) contains supplementary material, which is available to authorized users.

## Introduction

Platform chemicals are the ones that serve as the basic starting materials for producing chemical intermediates, building block compounds, and polymers. Giving an example, ethylene is used as a building block compound for the production of polyethylene. Short chain diols, which are exemplified by ethylene glycol, propanediols (PDO), butanediols (BDO), are of great importance in the family of platform chemicals. Owing to their unique structures and reaction activities, they are widely used for the synthesis of polymers, special chemicals and chemical intermediates.

The chemical production of short chain diols from petroleum materials has been developed and optimized for decades [1]. For example, Reppe chemistry used acetylene for BDO production representing the largest portion of this field [2]. However, the chemical syntheses methods of diols usually require high pressure, high temperature, expensive catalyzers, release of toxic intermediates, dependence on non-renewable materials, which result in complex process and low yield [3]. The development of environmentally benign, or “green”, processes for the production of chemicals is of increasing importance as nonrenewable resources are depleted and world population grows. Microbial fermentation processes are particularly attractive in this regard because they typically use renewable feedstocks such as glucose or sucrose and do not generate toxic byproducts.

Currently, a variety of biotechnologies have been developed for the microbial production of short chain diols. Previous articles have individually or partially reviewed the biotechnological production of 1,3-PDO, 1,2-PDO, 1,4-BDO or 2,3-BDO [4]-[7]. In this review, the whole processes of bio-diols production were summarized and discussed, from upstream process including substrate, microorganism, metabolic pathway and fermentation process, to downstream process. Furthermore, the research progress and potential biosynthesis method of pentanediols were discussed in this paper.

### Substrate

For the microbial production of diols the substrate cost can make up much of the whole producing cost. Glucose is widely used in fermentation processes. DuPont and Genencor Inc. have successfully developed the engineered *E. coli* to produce 1,3-PDO from glucose which represents the state-of-the art [8]. The price of glucose is a little expensive for large-scale commercial production. Glycerol is an inevitable byproduct generated during both bioethanol and biodiesel production processes, and the tremendous growth of these industries has led to a dramatic decrease in crude glycerol prices over the past few years [9]-[11]. Studies showed that raw glycerol can be used for diol production. Crude glycerol was converted to 1,3-PDO by a newly isolated *Citrobacter freundii* strain FMCC-B 294 (VK-19) with a relatively high conversion yield of 0.36 g/g [12]. A mutant strain of *Clostridium pasteurianum* appeared great tolerant to crude glycerol and in a fermentation, the productivity of 1,3-PDO was 1 g/l/h with a yield of 0.21 g/g [13]. An engineered *E. coli* strain produced S-1,2-PDO aerobically from crude glycerol with a titer of 0.44 g/L [14]. Production of 2,3-BDO was achieved using crude glycerol by an engineered *E. coli* [15].

For further reduce the cost, some more cheaper and abundant substrates, which include renewable agricultural resources (starch), food industry residues (starch hydrolysate, molasses and whey permeate) and lignocellulosic biomass (wood hydrolysate) have a distinguished edge. Several studies on 2,3-BDO production from lignocellulosic biomass hydrolysates, such as straw paper pulp enzymatic hydrolysate and corncob acid hydrolysate, were reported in recent years [16]-[18]. For example, cellodextrin was used to produce 2,3-BDO with an engineered *E. coli*. At an approximately 24 h fermentation, the highest yield of BDO was 0.84 g/g [19].

Jerusalem artichoke is an inexpensive and readily available non-grain raw material. Fresh Jerusalem artichoke tubers contain nearly 20% (w/w) carbohydrates, in which there is 70–90% (w/w) inulin [20],[21]. Some strains of *Paenibacillus polymyxa* were proved to be capable of fermenting biopolymers such as inulin without previous hydrolysis [22]. For example, 37 g/L of (R,R)-2,3-BDO at more than 98% optical purity was achieved by a *P. polymyxa* ZJ-9 with a medium for one-step fermentation of raw inulin extract from Jerusalem artichoke tubers [23]. This process greatly decreased the raw material cost and thus facilitated its practical application. In a previous research, 2,3-BDO production from Jerusalem artichoke by *Bacillus polymyx*a ATCC 12321 was optimized in batch culture [24]. Additionally, Jerusalem artichoke tubers was also utilized for 2,3-BDO production by *Klebsiella pneumoniae* [25].

**Figure 1:**
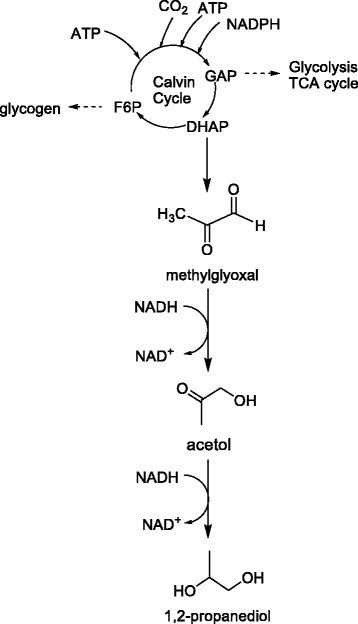
**Utilization of CO**_**2**_ **for 1,4-BDO production. GAP: glyceraldehyde-3-phosphate; F6P: fructose-6-phosphate; DHAP: dihydroxyacetone phosphate.**

Besides above mentioned substrates, CO_2_ is used to synthesize 1,2-PDO. A latest study reported the photosynthetic production of 1,2-PDO from CO_2_ using a genetically engineered cyanobacterium *Synechococcus elongatus* PCC 7942. The synthetic pathway is shown in Figure 1. The best engineered *S. elongatus* strain LH22 introducing *mgsA* and *yqhD* both from *E. coli* and the *adh* from *Clostridium beijerinckii* produced ~150 mg/L 1,2-PDO [26]. Direct 1,2-PDO production from CO_2_ in photosynthetic organisms recycles the atmospheric CO_2_ and will not compete with food crops for arable land, compared to the previously reported biological production processes using sugar or glycerol as the substrates. Even though this method has advantages like environmentally friendly and low cost, it is essential to further improve yield and productivity increasing the competitiveness.

### Microorganism: wide and engineered strains

#### 1,3-PDO

A number of microorganism species are able to accumulate short chain diols. 1,3-PDO can be produced by wide type strains like *Citrobacter* sp., *Clostridium* sp. and *Klebsiella* sp.. *Citrobacter freundii* strain FMCC-B 294 (VK-19) was revealed to convert crude glycerol to 1,3-PDO [12],[27]. It has the ability to tolerate highly elevated amount of crude glycerol and 68.1 g/L of PDO were produced under fed-batch fermentation [27]. *Citrobacter amalonaticus* Y19 was investigated about 1,3-PDO production, and final yield reached 0.20 g/g under anaerobic conditions using fumatate and CoCl_2_ as electron acceptors [28]. The 1,3-PDO production potential of *Clostridium saccharobutylicum* NRRL B-643 was revealed in a previous study [29]. This strain was able to consume feed glycerol almost entirely, which made it to be a promising type for further 1,3-PDO studies. A mutant strain of *Clostridium pasteurianum* was obtained by random mutagenesis mediated using ethane methyl sulfonate to tolerate high concentrations of crude glycerol [13]. *Klebsiella pneumoniae* is a suitable biocatalyst for the production of 1,3-PDO, but limited by poor growth characteristics and higher pathogenic. A new *Klebsiella pneumoniae* J2B was isolated to produce 1,3-PDO, which showed better growth properties, lower lipopolysaccharide production, better ability to cope with the inhibitory effects of toxic reductive metabolites, and a higher sedimentation rate [30].

However, some efficient microbial producers like *Klebsiella* sp*.* are classified as class 2 (or pathogenic), thus not suitable for large scale industrial applications. The native 1,3-PDO producers from the lactobacilli group generally recognized as safe are potential biocatalysts for 1,3-PDO production. *Lactobacillus reuteri*, *Lactobacillus brevis* and *Lactobacillus bucheneri* have been reported to produce 1,3-PDO from mixtures of glucose and glycerol [31],[32]. Recently, *Lactobacillus diolivorans* was reported to reach product concentrations up to 86 g/L coupled with the addition of Vitamin B_12_ to the culture medium [33]. The max yield was 0.71 g/g when using 0.05 mol _glucose_/mol _glycerol_ without acetic acid in the batch medium. Another native producer, *Lactobacillus panis* strain (designated as PM1), was isolated from a stored bioethanol thin stillage sample [34]. It is a promising producer for industrial application with the high tolerance for salts and organic acids, and a wider tolerance for pH settings. Recent development in engineering of 1,3-PDO production strains are not covered in details in this paper, which are referred to representative reviewer [7].

#### 1,2-PDO

1,2-PDO can be produced by some natural producers like *Thermoanaerobacterium thermosaccharolyticum* [35], *Clostridium thermosaccharolyticum* [36] and *Clostridium sphenoides* [37]. *Thermoanaerobacterium thermosaccharolyticum*, has some properties such as being able to ferment a variety of renewable sugars and being thermophilic so that it can be seen as a potential host. Though 1,2-PDO production have been achieved using *T. thermosaccharolyticum*, the 1,2-PDO synthesis of this bacterium is not well understood. While *Corynebacterium glutamicum* has high potential as a commodity chemicals producer coupled with the prevailing abundant knowledge of its metabolism, this could imply the possibility of a very favorable host for 1,2-PDO production. Overexpressing *mgs* and *cgR_2242*, one of the genes annotated as aldo-keto reductases (AKRs) in *C. glutamicum*, enhanced the 1,2-PDO production to 25 mM compared with the 93 μM of wild-type strain after 132 h incubation under aerobic conditions [38].

Constructing benign *E. coli* catalysts for the production of PDO could be beneficial to commercial production because *E. coli* is well studied in metabolic mechanism, low cost and easy to culture. An enantiomerically pure R-1,2-PDO was produced from glucose in an engineered *E. coli* and 0.7 g/L 1,2-PDO was obtained by co-expressing methylglyoxal synthase and glycerol dehydrogenase [39]. A further improvement of 1,2-PDO production in *E. coli* was conducted through eliminating production of a byproduct, lactate, constructing a complete pathway to 1,2-PDO from DHAP and investigating bioprocessing improvements of a fed-batch fermentation. Finally, a titer of 5 g/L of (*R*)-1,2-PDO was produced, with a final yield of 0.19 g of 1,2-PDO per gram of glucose consumed [40]. An engineered *E. coli* strain was reported to produce 5.6 g/L 1,2-PDO from glycerol at a yield of 0.21 g/g [41]. The advantage of this strain is that the titer and yield achieved were favorable to those obtained with the use of *E. coli* for the 1,2-PDO production from common sugars.

*Saccharomyces cerevisiae* is also a widely used genetic engineering bacteria. *Saccaromyces cerevisiae* strains have been used as 1,2-PDO producer for its higher stability than that of prokaryotes [42]-[44]. An engineered *S. cerevisiae* harboring the *mgs* gene of *E. coli*-K12 MG1655 and the *dhaD* gene of *Citrobacter freundii* achieved 0.45 g/L 1,2-PDO in a batch fermentation [42]. The distinguished advantage of this strain was the increased extracellular concentration yield of 1,2-PDO because the external discharge capacity of a cell membrane and the processes of releasing bio-metabolites from a cell membrane determined the economic and utility values of bio-metabolites. Another engineered *S. cerevisiae* strain was constructed by expressing the *E. coli mgs* and *gldA* genes, which encode methylglyoxal synthase and glycerol dehydrogenase, respectively [44]. This study evaluated the effect of *mgs* and *gldA* gene number on 1,2-PDO production showing that the strain containing 3 copies produced the highest level of 1,2-PDO. An recombinant *S. cerevisiae* was constructed by engineering both glycerol dissimilation and 1,2-PDO pathways. With these strategies, glycerol utilization and grow rate were increased [45]. This is of great importance because the poor growth rate and glycerol utilization ability of wide strain made it difficult to use glycerol as carbon source.

#### 2,3-BDO

Species noted for its 2,3-BDO producing ability belong to the genera *Klebsiella*, *Enterobacter*, *Bacillus* and *Serratia*. Native producers, such as *Klebsiella pneumoniae* [46], *Klebsiella oxytoca* [47] and *Bacillus polymyxa* [48], have been used for BDO production. However, the pathogenicity of opportunistic infection caused by the *Klebsiella* species is generally thought to be an obstacle hindering the large-scale 2,3-BDO production. *Bacillus* and *Paenibacillus polymyxa* has been reported to produce R,R-2,3-BDO [24],[49],[50]. A newly developed thermophilic *Bacillus licheniformis* was reported to convert glucose and xylose to D-(-)-2,3-BDO at 50°C with the optical purity more than 98% [51]. As a high temperature producer matching the simultaneous saccharification and fermentation conditions, it has potential to further lower BDO producing cost with low cost lignocellulosic biomass. *Enterobacter cloacae* NRRL B-23289, isolated from local decaying wood/corn soil samples, demonstrated a 2,3-BDO yield of 0.4 g/g arabinose with a corresponding productivity of 0.63 g/l/h. The bacterium has the capability to convert multiple mixed sugar substrates to 2,3-BDO thus it may be appropriate for developing the commercial production of 2,3-BDO from corn fiber and other agricultural residues [52].

Strain improvement for efficient microbial 2,3-BDO production involves not only native producers, i.e. homologous hosts, but also microorganisms with acquired ability to form 2,3-BDO via genetic manipulations, i.e. heterologous hosts. The *lactic acid bacteria* (LAB) are potential alternatives to produce 2,3-BDO with its safety and possession of a natural 2,3-BDO biosynthetic pathway [53]. An engineered *Laclobacillus plantarum* was constructed through elimination of lactate dehydrogenase activity and expression of butanediol dehydrogenase activity, and 0.49 g/g of 2,3-BDO yield was claimed in this engineered strain [54]. *Serratia marcescens* H30 was reported as a potential strain to produce 2,3-BDO from sucrose [55]. However, *Serratia marcescens* produces serrawettin W1, a surface-active exolipid, which will result in a lot foam formation leading to microbial contamination and bacterial activity decrease during the 2,3-BDO fermentation process. In order to overcome this drawback, mutants deficient in serrawettin W1 formation were constructed through insertional inactivation of the *swrW* gene coding for serrawettin W1 synthase [56]. The mutant strain afforded a maximum 2,3-BDO concentration of 152 g/L with a productivity of 3 g/l/h and yield of 0.93 g/g during a fed-batch culturing.

*E. coli* is frequently used as a heterologous host engineered for enantio-pure 2,3-BDO production. In a report, meso-2,3-BDO was produced by an engineered *E. coli* introduced two biosynthesis genes - *budA*, encoding acetolactate decarboxylase, and *meso*-*budC*, encoding meso-secondary alcohol dehydrogenase from *Klebsiella pneumonia* [15]. Optically pure meso-2,3-BDO (purity >99%) was produced at high yield of 0.31 g/g and 0.21 g/g from glucose and crude glycerol feed, respectively, under optimized conditions. There is also another engineered *E. coli* capable of producing meso-2,3-BDO from cellodextrin with a yield of 0.84 g/g [19]. The strain was constructed by (i) deleting gene including *poxB* and *ackA-pta*, (ii) introducing a synthetic operon which consists of genes encoding acetoacetate synthase, acetoacetate decarboxylase and secondary alcohol dehydrogenase, (iii) periplasmic expressing a *Saccharophagus* cellodextrinase. *E. coli* was used to produce acetoin, a precursor of 2,3-BDO [57]. The research could make a guidance for 2,3-BDO industrial biosynthesis.

#### 1,4-BDO

Though there are not a large amount of reports about 1,4-BDO microbial production, now, through the system-wide metabolic engineering, rational protein engineering and genetic engineering strategies, an engineered *E. coli* was capable to directly produce 18 g/L 1,4-BDO from renewable carbohydrate feedstock [58]. In addition, the toxicity of 1,4-BDO is reckoned as a key factor restricting its industrial production. So 1,4-BDO tolerant strains are under research. A biosensor, which detected 1,4-BDO using a fluorescent gene under the control of the *yhjX* (a predicted major facilitator superfamily protein) promoter, was developed on the foundation of the large differential expression of *yhjX* in response to 1,4-BDO, which provided a powerful tool to research the 1,4-BDO production strains [59].

### Other short chain diols

Various microorganisms used for diols production are summarized in Tables 1 and 2. There are less reported researches on other butanediols except 2,3-BDO, especially 1,3-BDO and 1,2-BDO. But 1,3-BDO is expensive than 2,3-BDO (Table 3), exploring its biosynthesis technology will have broad market value. Some metabolic intermediates of glycolysis and TCA cycle are used in existing biosynthetic pathways of 2,3-BDO and 1,4-BDO, however, there aren’t precursors or structural analogs for 1,3-BDO and 1,2-BDO in the major metabolic pathways, therefor this defect restricts their microbial production. Up to mow, the microbial productions of 1,3-BDO have been reported in patents by engineered microbial organisms [60],[61]. Alanine, acetoacetyl-CoA and 4-hydroxybutyryl-CoA were used as substrates through non-natural metabolic pathways. Though microbial production of 1,2-BDO isn’t reported, the existing BDO non-natural microbial production methods provide guidance for its development.

**Table 1:** **Wide strains for diols production**

Strains	Substrate	Methods	Productivity/Yield	Reference
1,3-Propanediol				
*Lactobacillus reuteri*ATCC 23272	Glucose and glycerol	Co-fermentation	1 g/l/h	[62]
*Lactobacillus panis* PM1	Glycerol or/and Glucose	-	-	[34]
*Klebsiella pneumoniae* J2B	Glycerol	Fed-batch fermentation	0.15 g/l/h	[30]
*Klebsiella pneumoniae* CGMCC 2028	Glycerol	Continuous fermentation	0.43 g/g	[63]
*Citrobacter freundii* DSM 15979	Crude glycerol	Packed bed biofilm reactors operating under continuous conditions	-	[64]
*Pantoeaagglomerans DSM 30077*		Packed bed biofilm reactors operating under continuous conditions	4 g/l/h PUF^*^	[64]
*Citrobacteramalonaticus* Y19	Glycerol	anaerobic conditions using fumatate and CoCl_2_ as electron acceptors	0.20 g/g	[28]
*Citrobacterfreundii* FMCC-B 294(VK-19)	waste glycerol	Fed-batch fermentation	0.79 g/l/h	[27]
*Clostridium butyricum* VPI 3266	Glycerol	Continuous cultures	-	[65]
*Clostridium butyricum* VPI 1718	Glycerol	Batch and continuous cultures	0.56 g/g (batch culture)	[66]
			0.57 g/g (continuous culture)	
*Clostridium butyricum* F2b	Industrial glycerol	Batch and continuous cultures	6 g/ l/h (max)	[67]
*Clostridium saccharobutylicum* NRRL B-643	Glycerol	-	0.30 g/g	[29]
*Clostridium beijerinckii* NRRL B-593	Raw glycerol	Immobilized bioreactor continuous operation	0.78 g/g (ceramic rings)	[68]
			0.79 g/g (pumice stones)	
2,3-Butanediol				
*Paenibacilluspolymyxa*	Raw inulin extract from Jerusalemartichoke tuber	Batch fermentation	0.88 g/l/h	[23]
2,4-Pentanediol				
*Candida boidinii* KK912	2,4-Pentanedione	-	-	[69]

^*^PUF: polyurethanefoam, a material packed in column bioreactors.

**Table 2:** **Engineered strains for diols production**

Strains	Genes/Enzymes	Substrate	Methods	Productivity/Yield	Reference
**1,3-Propanediol**					
*Clostridium acetobutylicum* DG1(pSPD5)	Glycerolkinase, Glycerol-3-phosphate dehydrogenase	Glycerol	Continuous cultures	-	[65]
Recombinant *E. coli*	Glycerol3-phosphate dehydrogenase, glycerol 3-phosphate phosphatase, Insert *dhaB1-3*, *yqhD*, Delete *tpi*	Glucose	Fed-batch fermentation	4 g/l/h	[8]
**1,2-Propanediol**					
*Escherichia coli*	Methylglyoxalsynthase, Glycerol dehydrogenase, Aldehyde oxidoreductase, ATP-dependentdihydroxyacetone kinase	Glycerol	Multi-fermentation	0.21 g/g	[41]
*Saccharomyces cerevisiae*(sJDPMG)	Overexpress genes:*gdh*,*GUP1, mgs*, *gldA*	Glycerol	Flask cultivation	0.21 ± 0.02 g/g	[45]
*Synechococcus elongates* PCC 7942	*mgsA*, *yqhD*, *adh*	CO_2_	Light condition	--	[26]
*Corynebacterium glutamicum*	Overexpress genes:*mgs*, *cgR_2242*	Glucose	Flask cultivation	20 mg/l/h	[38]
*Pichia pastoris*	Introduce genes:*mgsA*, *yqhD*, *glydh*	Glycerol	Fed-batch fermentation	2 mg/l/h	[70]
**2,3-Butanediol**					
*Serratiamarcescens*H303	*swrW*	Sucrose	Fed-batch culturing	3 g/l/h	[56]
*Bacillus licheniformis*	Knock out the*ldh*	Glucose/Xylose	Anaerobic fermentation	0.45 g/g (BL5 glucose), 0.44 g/g(BL8 xylose)	[51]
*Escherichia coli* SGSB03	Introduce genes: *budA*, *meso-budC*	Glucose/Glycerol	Batch culture	0.31 g/g (glucose)	[15]
				0.21 g/g (glycerol,)	
*Escherichia coli* MGLAP	Acetoacetate synthase, Acetoacetatedecarboxylase, Secondary alcohol dehydrogenase	Cellodextrin	Anaerobic cultivation	0.84 g/g	[19]
1,4-Butanediol					
*Escherichia coli*	Knock out genes: *adhE*, *pflB*, *ldhA*, Replace *lpdA*		Fed-batchfermentation	0.16 g /l/h	[58]

**Table 3:** **Some main details about diols**

Diols	Boiling point	Applications	Price $/ton	Cost* $/ton
1,3-propanediol	210-211°C	a building block of polymers; antifreeze in wood paint.	1500-1600	774
1,2-propanediol	188.2°C	unsaturated polyester resins; used in the oil dispersant; environmentally friendly automotive antifreeze;	900-1400	774
2,3-butanediol	183-184°C	ingredients of methylethylketone; a precursor of chiral carriers of drug; a flavouring agent	1000-5000	1300
1,4-butanediol	228°C	elastic fibers and polyurethanes; a recreational drug; the synthesis of γ-butyrolacton	2200-2500	-
1,3-butanediol	207.5°C	a humectant and softener; a material for liquid crystals	4000-5000	-

*Cost represents the estimated price which is calculated according to the direct cost of the substrate (glucose).

There are rarely reports on the microbial productions of diols which have carbon chain above C4. And just 2,4-pentanediol, which plays an important role as chiral synthons for naturally occurring bioactive substances in the industrial field, has a synthesis report using a biocatalyst. A previous communication revealed the production of (2R,4R)-2,4-pentanediol with *Candida boidinii* RAMIREZ KK912 by the enantio-selective reduction of 2,4-pentanedione and stereo-inversion of the isomeric mixture of 2,4-pentanediol [69].

The carbon chain of pentanediols is longer, which possibly has a negative effect on microbial production. For further development, utilization of pentose sugar like xylose or C5 intermediates of metabolic pathways like citrate and α-ketoglutarate is likely to be a promising method. Zheng et al. realized microbial production of C5 monohydric alcohol, which provides an interesting perspective for C5 diols [71]. For example, through appropriate selection or manipulation P450 monooxygenase can be used for hydroxylating C5 monohydric alcohol to C5 diols.

### Metabolic pathway

#### 1,3-PDO

The metabolic pathways of 1,3-PDO production are mainly concentrated on two routes. One pathway can produce 1,3-PDO from glycerol. The glycerol pathway involves two enzymes - glycerol dehydratase which catalyzes the dehydration of glycerol to 3-hydroxypropionaldehyde, and 1,3-PDO dehydrogenase which subsequently converts the 3-hydroxypropionaldehyde to 1,3-PDO. 1,3-PDO dehydrogenases are NADPH- or NADH-dependent, like *DhaT* from *Klebsiella pneumoniae* and *YqhD* from *E. coli* [72]. The other pathway is starting from monosaccharide or polysaccharide substrates, producing 1,3-PDO from glucose, corn hydrolysate or sugarcane molasses. No known natural organism ferments sugars directly to 1,3-PDO. The attempts using sugars as substrate have been made with either multistage fermentations involving a combination of microorganisms [73],[74] or genetically engineered microorganisms [8],[75] carrying genes for the conversion of sugar to glycerol and then to 1,3-PDO.

**Figure 2:**
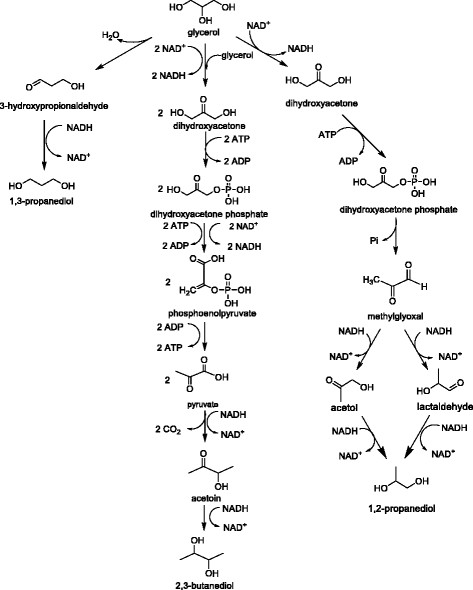
**Glycerol metabolism pathways of 1,3-PDO, 1,2-PDO and 2,3-BDO production.**

The corresponding reactions of 1,3-PDO production are summed up as below. O_2_ is required in glucose production route. This indicates that dissolved oxygen concentration will have great impact on the 1,3-PDO production. In glycerol route, growth occurs by the oxidative consumption of glycerol to produce cell mass concomitant with the accumulation of by-products (e.g. acetate) and the generation of excess NADH when oxygen is absent. The NADH cannot accumulate and must be converted to NAD^+^ so as to maintain a steady-state concentration of NADH and NAD^+^ within the cell. Thus, NAD^+^ is regenerated by the reductive conversion of glycerol to 1,3-propanediol in two steps as shown in Figure 2.

Glycerol:

$$C_{3}H_{8}O_{3}\to \;0.875C_{3}H_{8}O_{2}+0.375{CO}_{2}+0.5H_{2}O$$

Max Yield: 0.72 g/g

Glucose:

$$C_{6}H_{12}O_{6}+0.18O_{2}\to 1.45C_{3}H_{8}O_{2}+1.64{CO}_{2}+0.18H_{2}O$$

Max Yield: 0.61 g/g

**Figure 3:**
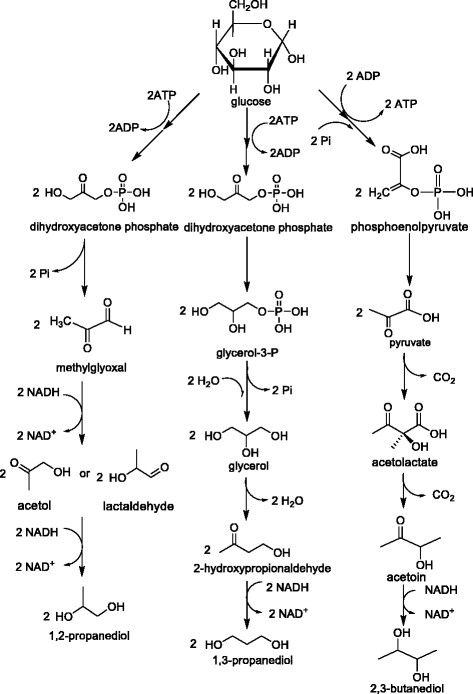
**Glucose metabolism pathways of 1,3-PDO, 1,2-PDO and 2,3-BDO production.**

The maximum theoretical yield of 1,3-PDO from glycerol is clearly higher than that from glucose. It is clear that glucose is more demanding in terms of stoichiometric requirements for balance. What’s more, seeing from figures (Figures 2 and 3), the glucose pathway is indeed via glycerol, then to produce 1,3-PDO. Considering the cost, glycerol, a by-product of biodiesel, is a competitive substrate for 1,3-PDO production on account of its low cost, abundance, and the higher degree of reduction.

Though the oxygen is not needed in the glycerol reaction, low amounts of oxygen enable a better growth of the culture and therefore increase the overall number of biocatalysts for the conversion of glycerol to 1,3-PDO and thus increasing the 1,3-PDO production. Vitamin B12 is the co-factor of most glycerol dehydratases. It was reported that the level of 1,3-PDO production was improved when vitamin B12 was added to the culture medium [28]. So it is likely that an efficient synthesis of Vitamin B12 by the cell is helpful to maintain a high level of activity of glycerol dehydratase then to improve 1,3-PDO production.

#### 1,2-PDO

In 1,2-PDO production, glucose is the most widely used sugar substrate [76]-[78]. Glucose undergoes glycolysis and enters the 1,2-PDO pathway by dihydroxyacetone phosphate (DHAP) which is metabolized to methylglyoxal (MG). From MG, two pathways, lactaldehyde-forming and acetol-forming pathway, are found to produce 1,2-PDO [70]. Owing to the wide availability, low-price, and high degree of reduction, glycerol is an attractive carbon source for the production of 1,2-PDO. In contrast to the 1,3-PDO pathways that convert glycerol directly to diols in two-steps, the typical fermentative pathways for 1,2-PDO production from glycerol require the conversion of the carbon source to DHAP through glycolytic pathways, which requires that the organism of interest possesses the ability to utilize glycerol directly [79]. The glycerol pathway and glucose pathway are respectively summarized in Figures 2 and 3.

The best possible yield of 1,2-PDO is obtained from a simple stoichiometric balance without consideration of metabolic pathways or energetics, for example, the yield from glucose is 0.84 g/g. In actual fermentation, more oxidized co-products must be produced to maintain an overall electron balance. Furthermore, sufficient energy in the form of ATP must be generated to enable the fermenting organisms to grow and remain viable. However, this yield obtained from a simple stoichiometric balance indicates that a high-yielding photochemical or electro-chemical route to PDO may be possible in the future.

Considering the metabolic pathways and energetics, the corresponding reactions of 1,2-PDO production are summed up as below. Both routes need oxygen. Though the max yields of these two reactions are comparable, glycerol pathway (Figure 2) requires less NADH and ATP compared with glucose pathway (Figure 3). In addition, the glycerol pathway has fewer steps, thus this makes the metabolic engineering easy to operate on this pathway.

Glycerol:

$$C_{3}H_{8}O_{3}+0.23O_{2}\to \;0.82C_{3}H_{8}O_{2}\;+0.56{CO}_{2}+0.73H_{2}O$$

Max Yield: 0.67 g/g

Glucose:

$$C_{6}H_{12}O_{6}+\;0.18O_{2}\;\to \;1.45C_{3}H_{8}O_{2}+1.64{CO}_{2}+0.18H_{2}O$$

Max Yield: 0.61 g/g

There is still a gap between the actual yield and the theoretical one so that different cultivation parameters, such as oxygen supply, feeding-strategy, or medium composition are deserved to be applied in actual fermentation. Fed-batch fermentation and multi-fermentation is effective in product increase. The co-fermentation of glucose and glycerol in some organisms like *Lactobacillus diolivorans* leads to a shift from NADH-consuming to NADH-producing reactions during glucose catabolism. This indicates a promising aspect in improvement to product yield.

#### 2,3-BDO

The biosynthesis pathways of 2,3-BDO using glycerol and glucose as substrates are respectively shown in Figures 2 and 3. In glucose production route, respiratory quotient is an important variable. Under microaerobic conditions, oxygen competes for NADH consumption among the lactate pathway and the ethanol pathway, thus the formation of lactate and ethanol would reduce. Under conditions of adequate oxygen supply, the TCA cycle is also active, which competes for the intermediate pyruvate with the 2,3-BDO pathway and supplies additional energy (ATP) and reducing equivalents, leading to a decreased formation of 2,3-BDO. Glycerol has a lower energy value compared with glucose and other 6-carbon carbohydrates, therefore, compared to glucose, glycerol is expected to result in lower yields of pyruvate-derived biochemical.

The corresponding reactions of 2,3-BDO production are summed up as below. The max yields of these two reactions are comparable. Glycerol pathway requires ATP, while glucose pathway produces ATP. Considering the energy consumption, glucose pathway is more advantageous.

Glycerol:

$$C_{3}H_{8}O_{3}+1.39O_{2}\to 0.47C_{4}H_{10}O_{2}+\;1.11{CO}_{2}+1.63H_{2}O$$

Max Yield: 0.46 g/g

Glucose:

$$C_{6}H_{12}O_{6}+2ADP+{NAD}^{+}\to C_{4}H_{10}O_{2}+2{CO}_{2}+2ATP+2H_{2}O+\mathrm{NADH}+H^{+}$$

Max Yield: 0.5 g/g

Compared with PDO productions, the utilization efficiency of carbon atom in 2,3-BDO production is obviously lower, no matter that the substrate is glycerol or glucose. The reason is the more release of CO_2_ in synthetic pathways, namely, the carbon loss. To improve efficiency, reuse of CO_2_ or carbon rearrangement avoiding the release of CO_2_ is likely to be an effective way [80].

#### 1,4-BDO

**Figure 4:**
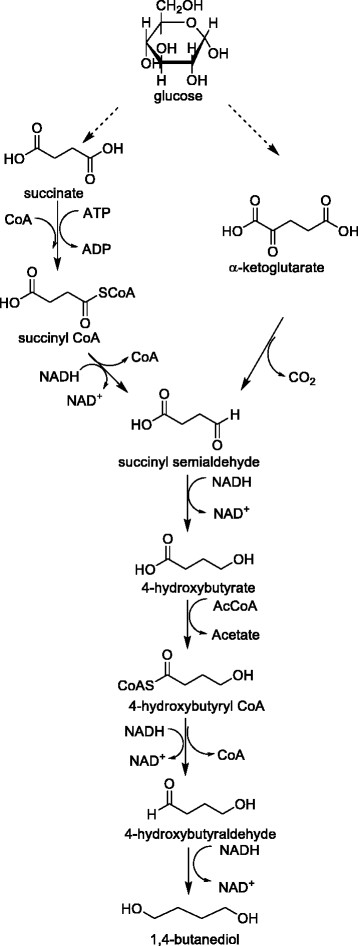
**Glucose metabolism pathways of 1,4-BDO production.**

1,4-BDO production pathways from glucose are shown in Figure 4. The non-naturally 1,4-BDO production was reported in patents [81]-[83]. In WO patent 2011047101, researchers demonstrated 1,4-BDO production pathways from succinate, succinyl-coenzyme A (CoA) and α-ketoglutarate [83]. Another invention provided a non-naturally occurring microbial biocatalyst to produce 1,4-BDO from 4-hydroxybutanoic acid with various pathways including the use of the native *E. coli* SucD enzyme to convert succinate to succinyl-CoA, and the use of α-ketoglutarate decarboxylase in the α-ketoglutarate pathway [81].

### Fermentation process

On an industrial scale, the production costs can be decreased by increasing the process productivity as well as shortening the time for upstream and downstream processes. Immobilization techniques can help to reach the above goal. There are some studies with immobilized cell systems for PDO production. *Clostridium beijerinckii* was reported to produce 1,3-PDO using raw glycerol in immobilized cell system [68]. And yields were 0.78 g/g and 0.79 g/g, respectively, when ceramic rings and pumice stones were used as immobilization supports. When using *Klebsiella pneumoniae* as producer for 1,3-PDO, ceramic-based cell immobilization achieved a higher production rate of 10 g/l/h in comparison with suspended cell system 4.9 g/l/h. An immobilized *Klebsiella* sp. HE-2 cells were reported be used for six cycles without significant activity loss so as to greatly improve the operational stability and reusability of the cells [84].

Supporting material plays an important role in immobilization. Effective strain attachment to the packing material surface could improve the operational process. Gungormusler et al. revealed that ceramic materials were good candidates for immobilization purpose, and ceramic balls were better support materials than ceramic rings in terms of immobilization [85]. Zhao et al. investigated the potential of microcapsule of sodium cellulose sulfate/polydimethyl-diallyl-ammonium chloride (NaCS/PDMDAAC) in the immobilization of *K. pneumoinae* for 1,3-PDO production [86]. And a high 1,3-PDO productivity was up to 16.4 g/l/h, however, they used pure glycerol as the substrate. Other researchers revealed the feasibility of using a novel microbial strain and packing material, namely, *Pantoea agglomerans* and Vukopor®S10, respectively, in the bioconversion of crude glycerol into 1,3-PDO in packed bed biofilm reactors [64]. When using polyurethanefoam as a packing stock, the productivity was 4 g/l/h much higher than 1 g/l/h in suspended cell systems. What’s more, this strain demonstrated effective attachment to the packing material surface.

Most conventional fermentations are sterile processes. While the implement of non-sterile conditions minimizes the energy cost for sterilization, as well as the overall cost for equipment, various investigations were performed on non-sterile fermentation. 1,3-PDO fermentation can efficiently and successfully be performed under completely non-sterile fermentation conditions [87]-[89]. Microorganisms tested in these studies were strains of the species *Clostridium butyricum* and *Klebsiella oxytoca*. To find more species possessing this advantage, *Citrobacter freundii* was also cultured in non-sterilized fed-batch process, and 176 g/L of raw glycerol were converted to 66 g/L 1,3-PDO [27]. The absence of aseptic conditions proved to have no considerable effect on glycerol fermentation by *C. freundii*.

### Downstream recovery techniques

**Figure 5:**
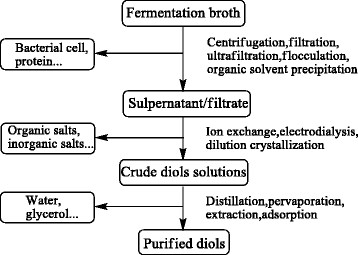
**Main procedures and methods in diols separation.**

Recovery and purification is a challenging aspect in the development of most fermentation processes, and is expected to also be the case for the short chain diols. For the cost effective biological production, a low energy required separation process with a high efficiency is essential. In recent years, a large amount of researches on 1,3-PDO, 1,2-PDO and 2,3-BDO separation have been reported. Because the properties of short chain dios are similar, existing recovery methods play a guiding role in researches. Figure 5 shows the main procedures and methods in diols separation.

### Traditional separation methods

Many traditional separation methods involving distillation [47], pervaporation [90], steam stripping [91] and reverse osmosis [92] were established for the downstream processing. However, all these diols have boiling points that are significantly higher than that of water. For example, at 760 mmHg, the boiling points of 1,2-PDO and 1,3-PDO are 187.4 and 214°C, respectively. Therefore, evaporation and distillation processes will require the removal of water from the PDO rather than the PDO from water.

Evaporation or vacuum distillation has been attempted for the recovery of 1,3-PDO but appeared unattractive and uneconomical [93],[94]. Besides much energy consumption, the broth required desalination before evaporation. And the desalination process of 1,3-PDO fermentation broth gave low product yields [95],[96]. In order to save energy, multi-stage evaporation was ever investigated [97]. In single stage, the energy consumption was 1.2 kg steam/kg evaporated, while it was only 0.5 in multi-stage evaporation.

Compared with the distillation, solvent extraction method has a series of advantages, such as high separation efficiency, great capacity of production, and low energy consumption, thus attracting much attention. But diols are strongly hydrophilic, and their densities are not sufficiently different from water, so standard liquid - liquid extraction couldn’t achieve efficient separation. Various novel extraction methods are described in the following text.

### Complexation extraction

The main processes of complexation extraction method are (i) solute in a solution for separation contacts with extractants which contain complexing agents (consisting of complexing agent, co-solvent, diluent), (ii) complexing agents react with the solute for separation forming complex, and (iii) the corresponding complex transfers to the extraction solvent phase to achieve separation [98],[99].

Extractants are of great importance in complexation processes. The recovery of 1,2-PDO from aqueous solution was investigated using extractants consisting of ion pairs of Aliquat 336 and phenylboronate in 2-ethylhexanol, toluene, o-xylene, or di-isobutyl ketone and the separation scheme was based on the complexation of cis-vicinal diols with organoboronates [100]. Researchers recovered 1,3-PDO from dilute solution by complexation extraction using different complexing agents including tributyl phosphate, hexanoic acid, and octanoic acid [101].

Complexation extraction has advantages such as high selectivity and low cost. However, the distribution coefficients are not high so that this method is less efficiency. What’s more, the choice of specific extractant and suitable recovery system should be taken into consideration for each diol. Hence, the develop of novel extractant system and the recovery of extractants deserves more investigations to improve economic efficiency and product yield.

### Reactive extraction

In fermentation broth, the diols concentration is low. What’s more, they are highly hydrophilic, so converting diols to hydrophobic chemicals and then extracting with an appropriate solution may be an effective separation way.

Reactive extraction is an easy and energy-efficient process. Studiers proposed a reactive extraction method for 1,3-PDO recovery using formaldehyde or acetaldehyde to block the hydroxyl groups and aromatic hydrocarbons to recover corresponding intermediates by solvent extraction [102]. A UNIFAC program was used to select an appropriate solvent for the extraction of dioxane (the product of 1,3-PDO acetalization) [103]. A reactive extraction process using various aldehydes (propionaldehyde, butylaldehyde and isobutylaldehyde) as both the reactants and the extractants was also proposed in a research [104].

Besides reactants and extractants, catalysts also have impact on the reactive extraction. Two types of ion exchange resins, Dowex and Amberlite, were investigated for the reactive extraction of 1,3-PDO and the overall conversion of 1,3-PDO was 98% [105]. A low-cost sulfonated carbon-based catalyst was used for reactive extraction of 1,3-PDO from model fermentation mixture giving a conversion of 92% with 0.7 g catalyst/ 1 g 1,3-PDO [106]. ZrO_2_ − MoO_3_ solid heterogeneous catalyst was used in a reactive isolation approach for the recovery of 1,3-PDO from dilute aqueous solutions playing catalytic roles in both acetalization of 1,3-PDO and hydrolysis of 2-methyl-1,3-dioxane [107]. For ZrO_2_ − 10 wt % MoO_3_, the conversion rate of 1,3-PDO in acetalization reached 96%, and the acetals conversion in the hydrolysis reaction reached 97%. Reactive-extraction has also been used to separate 2,3-BDO from fermentation broth using sulfuric acid or hydrochloric acid as the catalyst [92],[108],[109].

Reactive extraction is an easy and energy-efficient process in which the reaction takes place simultaneously with the extraction. The process requires a shorter operating time and a smaller amount of reactant and extractant. But the recovery of reactant and extractant is inevitable which may increase the operation cost. In the future, finding more suitable reactants or catalysts and improving the properties of catalysts may be the focuses of researches.

### Salting-out extraction

Standard liquid-liquid extraction has been used to separate diols because of energy saving and simple process steps. However, the distribution coefficient of standard liquid-liquid extraction is not high in a dilute solution. Salting-out technology (the presence of inorganic salts decreasing aqueous solubility) was employed to overcome this obstacle. Salting-out extraction that employed a K_2_HPO_4_/ethanol system consisting of 21% ethanol and 17% K_2_HPO_4_ (mass fraction) was investigated to separate 2,3-BDO from fermentation broth and 99% of 2,3-BDO was recovered [110]. Some researchers studied the extraction of 2,3-BDO by adding different salts including KAl(SO_4_)_2_, (NH_4_)_2_SO_4_, K_2_HPO_4_, into solvent n-butanol. The results indicated that K_2_HPO_4_ was a promising salt with distribution coefficient of 5%, separation factor of 43% and recovery yield of 80% [111].

Salting-out extraction is also employed to recovery of 1,3-PDO. One-salt and two-salt systems were used to separate 1,3-PDO [112]. The organic solvent selected was hydroxyl (pentanol, hexanol, tributyl phosphate, 2-propanol, and isopropanol) rather than aldehyde solvent. Other researchers performed a continuous extraction of 1,3-PDO in a packed column using dipotassium phosphate/ethanol as salting-out extraction system [113]. During an 11 h continuous operation, 1,3-PDO recovery of 90% was obtained for the real fermentation broth.

Salting-out extraction has an improved distribution coefficient but it requires the choice of an appropriate salt. Moreover, the addition of salts not only increases the cost but also causes equipment corrosions.

### *In situ*product extraction

*In situ* product extraction during fermentation has been shown to be one of the advanced techniques that enhance the product recovery by reducing end-product effect.

A group of investigators tested the function of dealuminated NaY and Silicalite in separation of 1,3-PDO/aqueous solution. However, they didn’t study the selectivity of the glycerol [114]. Other researchers tested different kinds of X zeolite, Y zeolite and a Na-ZSM-5 zeolite founding that Na-ZSM-5 was better than X zeolite and Y zeolite [115]. However, salts were possible to percolate into adsorbents reducing separation efficiency. What’s more, the recovery of 1,3-PDO from the zeolite was not investigated.

Anvari and Khayati evaluated the feasibility of application of a series of selected solvents for the *in situ* extractive fermentation of *K. pneumoniae* in batch to overcome the accumulation effect of 2,3-BDO [116]. Results have clearly indicated that oleyl alcohol could be used as an alternative 2,3-BDO extractant. The effects of oleyl alcohol extraction on the 2,3-BDO formation by *K. pneumonia* were further investigated [117].

*In situ* product extraction reduces feedback inhibition; however, there are still some issues which need to be studied deeply, for example, the extraction efficiency of extractant, the reuse of extractant and the recovery of product from adsorbent.

### Aqueous two-phase extraction

Aqueous two-phase extraction (ATPE) has advantages like mild extraction condition, high capacity and easy realization of industrialization enlargement. The widely used aqueous two-phase systems (ATPSs) for purification are based on polyethylene glycol/salt systems or polymer/polymer systems.

Recently, short-chain alcohol/inorganic salt systems are attracting more attention owing to their advantages such as low cost, easy recovery of alcohol and simple scale-up [118]. This system has been used to purify natural compounds [119],[120], as well as diol separation.

An ethanol/ammonium sulfate aqueous two-phase system (APTS) was investigated to separate 1,3-PDO from fermentation broth [121]. This method was easy to realize industrial scale, because it could achieve the simultaneous separation of microbial cells and 1,3-PDO simplifying the separation process. However, methanol was used in the recycling of ammonium sulfate, which should be separated with ethanol. This separation process caused additional energy demand and inevitable alcohol lost. Considering the above disadvantages, another ATPS composed of methanol/phosphate where methanol could be effectively used both as extractant (for 1,3-PDO) and solvent (for crystallizing the salt) was suggested and studied in detail [122]. This method resolved the problems of alcohol loss and high energy expenditure caused by separating the extractant and the solvent for crystallizing salt. Under conditions of 35% (v/v) methanol, saturated concentration of phosphate and pH 10.7, the recovery of 1,3-PDO was 98%. Some researchers did a work to choose a suitable alcohol/salt ATPS and optimized with the response surface methodology [123].

The latest research progress on APTE is ionic liquid-based systems. Müller investigated the ability of water miscible ionic liquids to build ATPS for 1,3-PDO separation using 1-butyl-3-methylimidazolium trifluoromethansulfonate as the initial ionic liquid [124]. For an effective separation, there are some operational difficulties including the high phosphate concentration, waste stream requiring to be recycled and recovery of 1,3-PDO from the extract stream. An ionic liquid-based ATPS, phosphate/1-butyl-3-methylimidazolium trifluoromethanesulfonate (Im_4,1_CF_3_SO_3_)/water, was investigated in detail including the effects of the temperature, pH and Im_4,1_CF_3_SO_3_, phosphate and 1,3-PDO mass fractions on the phase diagram and the influence of the fermentation by-products on the liquid–liquid equilibrium [125]. Successively, researchers performed a continuously operated process in pilot-scale using the above mentioned aqueous two-phase system [126]. The PDO recovery for all simulated PDO mass fractions was nearly at 100% when using eight stages. Even though the extraction could be successfully performed, there are subjects including the investment cost, required space, and operational cost to be considered.

2,3-BDO and 1,3-PDO are similar in some properties. Researches on 1,3-PDO aqueous two-phase extraction has guidance for 2,3-BDO. Optimization of process variables for the 2,3-BDO extractive in ATPS and the phase system composition was studied using response surface methodology [127],[128]. A variety of APTSs were proposed including isopropanol/ammonium sulfate system [129], ethanol/phosphate system [130], and ethanol/ammonium sulfate system [131].

Aqueous two-phase extraction has advantages like mild extraction condition, high capacity and easy realization of industrialization enlargement. Additionally, ionic liquids have a variety of advantages including odorless, no pollution, non-flammable, separated from product easily, easy recovery and used repeatedly. Ionic liquid-based ATPE has a more competitive potential of economization and industrialization with its avoidance of serious problems including environment, health, safety, and equipment corrosion which are caused by the use of conventional organic solvents. Aqueous two-phase extraction is a promising method deserving more investigations in the future.

### Adsorption chromatography

The purification of 1,3-PDO was performed by the chromatographic purification method using various resins including DEAE cellulose, Amberlite, Dowex Monosphere and silica as stationary phase [132]. The yield of 1,3-PDO achieved was 89% with silica resin as stationary phase and a mixture of chloroform and methanol as eluent in gradient chromatography. In addition, advantages like simple equipment, easy maintenance and regeneration of silica gel made this method more promising.

In a recent study, newly developed chemically modified silica gels was examined for the recovery of 1,3-PDO from fermentation broth [133]. Through chemical modification, the mechanical strength of silica gels was improved and did not shrink or swell in organic solvents or aqueous solutions.

This method makes use of relatively simple equipment and easy maintenance. But the capacity of chromatography is not large enough to achieve industrial production.

### Technology-integrated separation

Integration of classical technologies and improvement or optimization of traditional technologies is an effective method to enhance separation efficiency. An integrated separation process of solvent extraction using 1-butanol as extracting solvent and pervaporation using polydimethylsiloxane membrane was used to recover 2,3-BDO from a synthetic fermentation broth [134]. In a later report, the ZSM-5-filled polydimethylsiloxane membrane was studied to separate 1-butanol/2,3-butanediol demonstrating good membrane selectivity and product purity [135]. Continuous efforts were made to study the process energy consumption in pervaporation and vacuum membrane distillation separation of 2,3-BDO [136].

An invention provided a 2,3-BDO production method that comprises anaerobic fermentation of a substrate containing CO_2_ to produce the compound and recovery of 2,3-BDO by a combination of ATPE and distillation after fermentation [137]. With several combined steps, the final concentration of the product was 98%. An integrated separation process based on reactive-extraction and reactive-distillation was developed in recovering 2,3-BDO from fermentation broth [138]. After a complete separation process, the yield of 2,3-BDO is more than 90% with purity over 99%. H_2_SO_4_ and HCl were used as catalyst of the separation process. Considering the equipment corrosion, they should be neutralized after reactive-distillation.

There are less reports on 1,4-BDO separation compared with 2,3-BDO. A recent invention disclosed several processes of isolating 1,4-BDO from the fermentation broth [139]. One process included using disc stack centrifugation and ultrafiltration to remove solids obtaining a liquid fraction, removing salts from the liquid fraction by evaporative crystallization and ion exchange, and distilling 1,4-BDO. In another embodiment, the solids were removed by disc stack centrifugation and ultrafiltration, remove of salts from the liquid fraction was achieved by nanofiltration and ion exchange, the final step was evaporation of water and distillation of 1,4-BDO.

Even though some properties of short chain diols are similar, each diol has its speciality so that reasonable separation methods are chosen according to the desire product. Give an example in reactive extraction, properties of different diols determine the choice of reactants and extractants. To summarize, methods and technologies studied so far have their advantages and drawbacks. Among the methods described above, aqueous two-phase extraction deserves more attentions.

## Conclusions

Bio-based production of short chain diols as platform chemicals is a promising method to substitute traditional petroleum production. For the glucose-based microbial production of diols, substrate costs make up much of the whole production costs. Raw glycerol from biodiesel production is a cheaper substrate and has been used for diols production. To further minimize substrate costs, farming and forestry residues like straw, Jerusalem artichoke tubers and wood hydrolysate deserve thoroughly investigation. The photosynthetic production of diols from CO_2_ using photosynthetic bacterium like cyanobacterium deserves attention in the future. In the area of microbial producers, beside engineering *E.coli* stains, *Klebsiella sp.* also is a suitable biocatalyst for an outstanding tolerance for high concentration glycerol, the ability to produce different diols and a high productivity (for example, a final 2,3-BDO yield close to 90% of theoretic value), but the pathogenicity should be overcome by engineering methods. In fermentation process, immobilization can result in a higher productivity and simplify recovery which shows the huge potential in commercial applications. Recovery of diols from fermentation broth is still an obstacle on commercial microbial production. In terms of yield and energy consumption, methods and technologies studied so far have their limitations or drawbacks. For further development, combination of classic separation techniques with other mew ones is essential. In conclusion, the integration of fermentation and downstream recovery help to fulfill cost efficient production process.

## Authors’ original submitted files for images

Below are the links to the authors’ original submitted files for images. Authors’ original file for figure 1 Authors’ original file for figure 2 Authors’ original file for figure 3 Authors’ original file for figure 4 Authors’ original file for figure 5

## Abbreviations

1: 2-PDO: 1,2-propanediol
1: 3-PDO: 1,3-propanediol
1: 2-BDO: 1,2-butanediol
2: 3-BDO: 2,3-butanediol
1: 3-BDO: 1,3-butanediol
1: 4-BDO: 1,4-butanediol
DHAP: Dihydroxyacetone phosphate
MG: Methylglyoxal
CoA: Coenzyme A
ATPE: Aqueous two-phase extraction
APTSs: Aqueous two-phase systems
APTS: Aqueous two-phase system

## References

[1] Haas T, Jaeger B, Weber R, Mitchell S, King C (2005). New diol processes: 1,3-propanediol and 1,4-butanediol. Appl Catal A-Gen.

[2] Cukalovic A, Stevens CV (2008). Feasibility of production methods for succinic acid derivatives: a marriage of renewable resources and chemical technology. Biofuels Bioprod Biorefining.

[3] Deckwer WD (1995). Microbial conversion of glycerol to 1,3-propanediol. FEMS Microbiol Rev.

[4] Bennett G, San KY (2001). Microbial formation, biotechnological production and applications of 1,2-propanediol. Appl Microbiol Biotechnol.

[5] Jang YS, Kim B, Shin JH, Choi YJ, Choi S, Song CW, Lee J, Park HG, Lee SY (2012). Bio-based production of C2-C6 platform chemicals. Biotechnol Bioeng.

[6] Willke T, Vorlop K (2008). Biotransformation of glycerol into 1,3-propanediol. Eur J Lipid Sci Technol.

[7] Zeng AP, Sabra W (2011). Microbial production of diols as platform chemicals: recent progresses. Curr Opin Biotechnol.

[8] Nakamura CE, Whited GM (2003). Metabolic engineering for the microbial production of 1,3-propanediol. Curr Opin Biotechnol.

[9] Yazdani SS, Gonzalez R (2007). Anaerobic fermentation of glycerol: a path to economic viability for the biofuels industry. Curr Opin Biotechnol.

[10] Khanal SK, Rasmussen M, Shrestha P, Van Leeuwen HJ, Visvanathan C, Liu H (2008). Bioenergy and biofuel production from wastes/residues of emerging biofuel industries. Water Environ Res.

[11] Rausch KD, Belyea RL (2006). The future of coproducts from corn processing. Appl Biochem Biotechnol.

[12] Metsoviti M, Paramithiotis S, Drosinos EH, Galiotou‐Panayotou M, Nychas GJE, Zeng AP, Papanikolaou S (2012). Screening of bacterial strains capable of converting biodiesel-derived raw glycerol into 1,3-propanediol, 2,3-butanediol and ethanol. Eng Life Sci.

[13] Jensen TØ, Kvist T, Mikkelsen MJ, Westermann P (2012). Production of 1,3-PDO and butanol by a mutant strain of *Clostridium pasteurianum*with increased tolerance towards crude glycerol. AMB Express.

[14] Mampel J, Meurer G, Eck J (2009). Production Method.

[15] Lee S, Kim B, Park K, Um Y, Lee J (2012). Synthesis of pure meso-2,3-butanediol from crude glycerol using an engineered metabolic pathway in *Escherichia coli*. Appl Biochem Biotechnol.

[16] Yang Y, Zhang Y, Sun Y, He L, Jiang Y (2010). Study on production of 2,3-butanediol from straw paper pulp hydrolysate fermentation by *Klebsiella pneumoniae*. Renew Energ Resour.

[17] Cheng KK, Liu Q, Zhang JA, Li JP, Xu JM, Wang GH (2010). Improved 2,3-butanediol production from corncob acid hydrolysate by fed-batch fermentation using *Klebsiella oxytoca*. Process Biochem.

[18] Jiang LQ, Fang Z, Guo F, Yang LB (2012). Production of 2,3-butanediol from acid hydrolysates of *Jatropha* hulls with *Klebsiella oxytoca*. Bioresour Technol.

[19] Shin HD, Yoon SH, Wu J, Rutter C, Kim SW, Chen RR (2012). High-yield production of meso-2,3-butanediol from cellodextrin by engineered *E. coli*biocatalysts. Bioresour Technol.

[20] Ge X, Zhang W (2005). A shortcut to the production of high ethanol concentration from Jerusalem artichoke tubers. Food Technol Biotechnol.

[21] Szambelan K, Nowak J, Czarnecki Z (2004). Use of *Zymomonas mobilis* and *Saccharomyces cerevisiae* mixed with *Kluyveromyces fragilis*for improved ethanol production from Jerusalem artichoke tubers. Biotechnol Lett.

[22] Celińska E, Grajek W (2009). Biotechnological production of 2,3-butanediol-current state and prospects. Biotechnol Adv.

[23] Gao J, Xu H, Li Q, Feng X, Li S (2010). Optimization of medium for one-step fermentation of inulin extract from Jerusalem artichoke tubers using Paenibacillus polymyxa ZJ-9 to produce R, R-2,3-butanediol. Bioresour Technol.

[24] Fages J, Mulard D, Rouquet JJ, Wilhelm JL (1986). 2,3-Butanediol production from Jerusalem artichoke, Helianthus tuberosus, by *Bacillus polymyx*a ATCC 12321. Optimization of k_L_a profile. Appl Microbiol Biotechnol.

[25] Sun LH, Wang XD, Dai JY, Xiu ZL (2009). Microbial production of 2,3-butanediol from Jerusalem artichoke tubers by *Klebsiella pneumoniae*. Appl Microbiol Biotechnol.

[26] Li H, Liao JC (2013). Engineering a cyanobacterium as the catalyst for the photosynthetic conversion of CO_2_to 1,2-propanediol. Microb Cell Fact.

[27] Metsoviti M, Zeng AP, Koutinas AA, Papanikolaou S (2012). Enhanced 1,3-propanediol production by a newly isolated *Citrobacter freundii*strain cultivated on biodiesel-derived waste glycerol through sterile and non-sterile bioprocesses. J Biotechnol.

[28] Ainala SK, Ashok S, Ko Y, Park S (2013). Glycerol assimilation and production of 1,3-propanediol by *Citrobacter amalonaticus*Y19. Appl Microbiol Biotechnol.

[29] Gungormusler M, Gonen C, Ozdemir G, Azbar N (2010). 1,3-Propanediol production potential of *Clostridium saccharobutylicum*NRRLB-643. New Biotechnol.

[30] Arasu MV, Kumar V, Ashok S, Song H, Rathnasingh C, Lee HJ, Seung D, Park S (2011). Isolation and characterization of the new *Klebsiella pneumoniae*J2B strain showing improved growth characteristics with reduced lipopolysaccharide formation. Biotechnol Bioprocess Eng.

[31] da Cunha MV, Foster MA (1992). Sugar-glycerol cofermentations in lactobacilli: the fate of lactate. J Bacteriol.

[32] Talarico TL, Axelsson LT, Novotny J, Fiuzat M, Dobrogosz WJ (1990). Utilization of glycerol as a hydrogen acceptor by *Lactobacillus reuteri*: purification of 1,3-propanediol: NAD+ oxidoreductase. Appl Environ Microbiol.

[33] Pflügl S, Marx H, Mattanovich D, Sauer M (2012). 1,3-Propanediol production from glycerol with *Lactobacillus diolivorans*. Bioresour Technol.

[34] Khan NH, Kang TS, Grahame DA, Haakensen MC, Ratanapariyanuch K, Reaney MJ, Korber DR, Tanaka T (2013). Isolation and characterization of novel 1,3-propanediol-producing *Lactobacillus panis*PM1 from bioethanol thin stillage. Appl Microbiol Biotechnol.

[35] Altaras NE, Etzel MR, Cameron DC (2001). Conversion of Sugars to 1,2-Propanediol by *Thermoanaerobacterium thermosaccharolyticum*HG-8. Biotechnol Prog.

[36] Cameron DC, Cooney CL (1986). A Novel Fermentation: The Production of R(-)-1,2-Propanediol and Acetol by *Clostridium thermosaccharolyticum*. Nat Biotechnol.

[37] Tran-Din K, Gottschalk G (1985). Formation of D(-)-1,2-propanediol and D(-)-lactate from glucose by *Clostridium sphenoides*under phosphate limitation. Arch Microbiol.

[38] Niimi S, Suzuki N, Inui M, Yukawa H (2011). Metabolic engineering of 1,2-propanediol pathways in *Corynebacterium glutamicum*. Appl Microbiol Biotechnol.

[39] Altaras NE, Cameron DC (1999). Metabolic engineering of a 1,2-propanediol pathway in *Escherichia coli*. Appl Environ Microbiol.

[40] Altaras NE, Cameron DC (2000). Enhanced production of (R)-1,2-Propanediol by Metabolically Engineered *Escherichia coli*. Biotechnology Prog.

[41] Clomburg JM, Gonzalez R (2011). Metabolic engineering of *Escherichia coli*for the production of 1,2-propanediol from glycerol. Biotechnol Bioeng.

[42] Jeon E, Lee S, Kim D, Yoon H, Oh M, Park C, Lee J (2009). Development of a *Saccharomyces cerevisiae*strain for the production of 1,2-propanediol by gene manipulation. Enzyme Microb Technol.

[43] Jung J-Y, Choi E-S, Oh M-K (2008). Enhanced production of 1,2-propanediol by tpi1 deletion in *Saccharomyces cerevisiae*. J Microbiol Biotechnol.

[44] Lee W, DaSilva NA (2006). Application of sequential integration for metabolic engineering of 1,2-propanediol production in yeast. Metab Eng.

[45] Jung JY, Lee JW (2011). Production of 1,2-propanediol from glycerol in *Saccharomyces cerevisiae*. J Bicrobiol Biotechnol.

[46] Ma C, Wang A, Qin J, Li L, Ai X, Jiang T, Tang H, Xu P (2009). Enhanced 2,3-butanediol production by *Klebsiella pneumoniae*SDM. Appl Bicrobiol Biotechnol.

[47] Afschar A, Vaz Rossell C, Jonas R, Quesada Chanto A, Schaller K (1993). Microbial production and downstream processing of 2,3-butanediol. J Biotechnol.

[48] Hespell R (1996). Fermentation of xylan, corn fiber, or sugars to acetoin and butanediol by *Bacillus polymyxa*strains. Curr Microbiol.

[49] De Mas C, Jansen NB, Tsao GT (1988). Production of optically active 2,3-butanediol by *Bacillus polymyxa*. Biotechnol Bioeng.

[50] Marwoto B, Nakashimada Y, Kakizono T, Nishio N (2002). Enhancement of (R, R)-2,3-butanediol production from xylose by Paenibacillus polymyxa at elevated temperatures. Biotechnol Lett.

[51] Wang Q, Chen T, Zhao X, Chamu J (2012). Metabolic engineering of thermophilic *Bacillus licheniformis*for chiral pure D-2,3-butanediol production. Biotechnol Bioeng.

[52] Saha B, Bothast R (1999). Production of 2,3-butanediol by newly isolated *Enterobacter cloacae*. Appl Microbiol Biotechnol.

[53] Gaspar P, Carvalho AL, Vinga S, Santos H, Neves AR (2013). From physiology to systems metabolic engineering for the production of biochemicals by Lactic Acid Bacteria. Biotechnol Adv.

[54] Paul B (2010). Enhanced Pyruvate to 2,3-Butanediol Conversion in Lactic Acid Bacteria.

[55] Zhang L, Yang Y, Sun JA, Shen Y, Wei D, Zhu J, Chu J (2010). Microbial production of 2,3-butanediol by a mutagenized strain of *Serratia marcescens*H30. Bioresour Technol.

[56] Zhang L, Sun JA, Hao Y, Zhu J, Chu J, Wei D, Shen Y (2010). Microbial production of 2,3-butanediol by a surfactant (serrawettin)-deficient mutant of *Serratia marcescens*H30. J Ind Microbiol Biotechnol.

[57] Jiang X, Zhang H, Yang J, Liu M, Feng H, Liu X, Cao Y, Feng D, Xian M (2013). Induction of gene expression in bacteria at optimal growth temperatures. Appl Microbiol Biotechnol.

[58] Yim H, Haselbeck R, Niu W, Pujol-Baxley C, Burgard A, Boldt J, Khandurina J, Trawick JD, Osterhout RE, Stephen R (2011). Metabolic engineering of *Escherichia coli*for direct production of 1,4-butanediol. Nat Chem Biol.

[59] Szmidt-Middleton HL, Ouellet M, Adams PD, Keasling JD, Mukhopadhyay A (2013). Utilizing a highly responsive gene, *yhjX*, in *E. coli*based production of 1,4-Butanediol. Chem Eng Sci.

[60] Burgard AP, Burk MJ, Osterhout RE, Pharkya P (2010). Organisms for the Production of 1,3-Butanediol.

[61] Burgard AP, Burk MJ, Osterhout RE, Sun J, Pharkya P (2012). Microorganisms for Producing 1,3-Butanediol and Methods Related Thereto.

[62] Baeza-Jiménez R, López-Martinez L, De la Cruz-Medina J, Espinosa-de-los-Monteros J, Garcıa-Galindo H (2011). Effect of glucose on 1,3-propanediol production by *Lactobacillus reuteri*. Revista Mexicana de Ingeniería Química.

[63] Wang Y, Teng H, Xiu Z (2011). Effect of aeration strategy on the metabolic flux of *Klebsiella pneumoniae*producing 1,3-propanediol in continuous cultures at different glycerol concentrations. J Ind Microbiol Biotechnol.

[64] Casali S, Gungormusler M, Bertin L, Fava F, Azbar N (2012). Development of a biofilm technology for the production of 1,3-propanediol (1,3-PDO) from crude glycerol. Biochem Eng J.

[65] González-Pajuelo M, Meynial-Salles I, Mendes F, Soucaille P, Vasconcelos I (2006). Microbial conversion of glycerol to 1,3-propanediol: physiological comparison of a natural producer, *Clostridium butyricum* VPI 3266, and an engineered strain, *Clostridium acetobutylicum*DG1 (pSPD5). Appl Environ Microbiol.

[66] Chatzifragkou A, Dietz D, Komaitis M, Zeng AP, Papanikolaou S (2010). Effect of biodiesel-derived waste glycerol impurities on biomass and 1,3-propanediol production of *Clostridium butyricum*VPI 1718. Biotechnol Bioeng.

[67] Papanikolaou S, Ruiz-Sanchez P, Pariset B, Blanchard F, Fick M (2000). High production of 1,3-propanediol from industrial glycerol by a newly isolated *Clostridium butyricum*strain. J Biotechnol.

[68] Gungormusler M, Gonen C, Azbar N (2011). Continuous production of 1,3-propanediol using raw glycerol with immobilized *Clostridium beijerinckii*NRRL B-593 in comparison to suspended culture. Bioprocess Biosyst Eng.

[69] Matsumura S, Kawai Y, Takahashi Y, Toshima K (1994). Microbial production of (2R,4R)-2,4-pentanediol by enatioselective reduction of acetylacetone and stereoinversion of 2,4-pentanediol. Biotechnol Lett.

[70] Barbier GG, Ladd JL, Campbell ER (2011). Genetic modification of *Pichia Pastoris*for production of propylene glycol from glycerol. Int J Genet Eng.

[71] Zheng Y, Liu Q, Li L, Qin W, Yang J, Zhang H, Jiang X, Cheng T, Liu W, Xu X (2013). Metabolic engineering of *Escherichia coli*for high-specificity production of isoprenol and prenol as next generation of biofuels. Biotechnol Biofuels.

[72] Johnson E, Lin E (1987). *Klebsiella pneumoniae*1,3-propanediol: NAD+ oxidoreductase. J Bacteriol.

[73] Hartlep M, Hussmann W, Prayitno N, Meynial-Salles I, Zeng AP (2002). Study of two-stage processes for the microbial production of 1,3-propanediol from glucose. Appl Microbiol Biotechnol.

[74] Mendes FS, González-Pajuelo M, Cordier H, François JM, Vasconcelos I (2011). 1,3-Propanediol production in a two-step process fermentation from renewable feedstock. Appl Microbiol Biotechnol.

[75] Tang X, Tan Y, Zhu H, Zhao K, Shen W (2009). Microbial conversion of glycerol to 1,3-propanediol by an engineered strain of *Escherichia coli*. Appl Environ Microbiol.

[76] Badía J, Ros J, Aguilar J (1985). Fermentation mechanism of fucose and rhamnose in *Salmonella typhimurium* and *Klebsiella pneumoniae*. J Bacteriol.

[77] Boronat A, Aguilar J (1979). Rhamnose-induced propanediol oxidoreductase in *Escherichia coli*: purification, properties, and comparison with the fucose-induced enzyme. J Bacteriol.

[78] Forsberg CW, Donaldson L, Gibbins L (1987). Metabolism of rhamnose and other sugars by strains of *Clostridium acetobutylicum* and other *Clostridium*species. Can J Microbiol.

[79] Clomburg JM, Gonzalez R (2012). Anaerobic fermentation of glycerol: A platform for renewable fuels and chemicals. Trends Biotechnol.

[80] Bogorad IW, Lin TS, Liao JC (2013). Synthetic non-oxidative glycolysis enables complete carbon conservation. Nature.

[81] Burk M, Van Dien S, Burgard A, Niu W (2008). Compositions and Methods for the Biosynthesis of 1,4-Butanediol and its Precursors.

[82] Burk M, Burgard A, Osterhout R, SUN J (2010). Microorganisms for the Production of 1,4-butanediol.

[83] Haselbeck R, Trawick J, Niu W, Burgard A (2011). Microorganisms for the production of 1,4-butanediol, 4-hydroxybutanal, 4-hydroxybutyryl-coa, putrescine and related compounds, and methods related thereto.

[84] Wong CL, Huang CC, Chen WM, Chang JS (2011). Converting crude glycerol to 1,3-propandiol using resting and immobilized *Klebsiella*sp. HE-2 cells. Biochem Eng J.

[85] Gungormusler M, Gonen C, Azbar N (2011). Use of ceramic-based cell immobilization to produce 1,3-propanediol from biodiesel-derived waste glycerol with *Klebsiella pneumoniae*. J Appl Microbiol.

[86] Zhao YN, Chen G, Yao SJ (2006). Microbial production of 1,3-propanediol from glycerol by encapsulated *Klebsiella pneumoniae*. Biochem Eng J.

[87] Temudo MF, Poldermans R, Kleerebezem R, van Loosdrecht M (2008). Glycerol fermentation by (open) mixed cultures: a chemostat study. Biotechnol Bioeng.

[88] Chatzifragkou A, Papanikolaou S, Dietz D, Doulgeraki AI, Nychas G-JE, Zeng AP (2011). Production of 1,3-propanediol by *Clostridium butyricum*growing on biodiesel-derived crude glycerol through a non-sterilized fermentation process. Appl Microbiol Biotechnol.

[89] Metsoviti M, Paraskevaidi K, Koutinas A, Zeng AP, Papanikolaou S (2012). Production of 1,3-propanediol, 2,3-butanediol and ethanol by a newly isolated *Klebsiella oxytoca*strain growing on biodiesel-derived glycerol based media. Process Biochem.

[90] Qureshi N, Meagher M, Hutkins R (1994). Recovery of 2,3-Butanediol by Vacuum Membrane Distillation∗. Sep Sci Technol.

[91] Garg S, Jain A (1995). Fermentative production of 2,3-butanediol: a review. Bioresour Technol.

[92] Xiu ZL, Zeng AP (2008). Present state and perspective of downstream processing of biologically produced 1,3-propanediol and 2,3-butanediol. Appl Microbiol Biotechnol.

[93] Ames TT (2002). Process for the Isolation of 1,3-Propanediol from Fermentation Broth.

[94] Kelsey DR (1996). Purification of 1,3-Propanediol.

[95] Gong Y, Tang Y, Wang X, Yu L, Liu D (2004). The possibility of the desalination of actual 1,3-propanediol fermentation broth by electrodialysis. Desalination.

[96] Hao J, Liu D (2005). Desalination of fermented broth containing 1,3-propanediol by Electrodialysis [J]. Chin J Process Eng.

[97] Hermann B, Patel M (2007). Today’s and tomorrow’s bio-based bulk chemicals from white biotechnology. Appl Biochem Biotechnol.

[98] Bi P, Dong H, Yu H, Chang L (2008). A new technique for separation and purification of l-phenylalanine from fermentation liquid: flotation complexation extraction. Sep Purif Technol.

[99] Black FJ, Bruland KW, Flegal AR (2007). Competing ligand exchange-solid phase extraction method for the determination of the complexation of dissolved inorganic mercury (II) in natural waters. Anal Chim Acta.

[100] Broekhuis RR, Lynn S, King CJ (1996). Recovery of propylene glycol from dilute aqueous solutions by complexation with organoboronates in ion-pair extractants. Ind Eng Chem Res.

[101] Xiang B, Chen S, Liu D (2001). Extraction of 1,3-propanediol in dilute fermentation broth. J Tsinghua University (Sci Technol).

[102] Broekhuis RR, Lynn S, King CJ (1994). Recovery of propylene glycol from dilute aqueous solutions via reversible reaction with aldehydes. Ind Eng Chem Res.

[103] Malinowski JJ (1999). Evaluation of liquid extraction potentials for downstream separation of 1,3-propanediol. Biotechnol Tech.

[104] Hao J, Liu H, Liu D (2005). Novel route of reactive extraction to recover 1,3-propanediol from a dilute aqueous solution. Ind Eng Chem Res.

[105] Malinowski JJ (2000). Reactive extraction for downstream separation of 1,3-propanediol. Biotechnol Prog.

[106] Boonoun P, Laosiripojana N, Muangnapoh C, Jongsomjit B, Panpranot J, Mekasuwandumrong O, Shotipruk A (2010). Application of sulfonated carbon-based catalyst for reactive extraction of 1,3-propanediol from model fermentation mixture. Ind Eng Chem Res.

[107] Wu M, Li C, Zhang J, Miao C, Zheng Y, Sun Y (2012). ZrO_2_–MoO_3_for the Acetalization of 1,3-Propanediol from Dilute Solutions. Ind Eng Chem Res.

[108] Hao J, Xu F, Liu H, Liu D (2006). Downstream processing of 1,3-propanediol fermentation broth. J Chem Technol Biotechnol.

[109] Li Y, Zhu J, Wu Y, Liu J (2013). Reactive-extraction of 2,3-butanediol from fermentation broth by propionaldehyde: Equilibrium and kinetic study. Korean J Chem Eng.

[110] Dai J, Zhang Y, Xiu Z (2011). Salting-out Extraction of 2,3-Butanediol from Jerusalem artichoke-based Fermentation Broth. Chin J Chem Eng.

[111] Wu YY, Chen K, Zhu JW, Wu B, Ji L, Shen YL (2014). Enhanced extraction of 2,3-butanediol by medley solvent of salt and n-butanol from aqueous solution. Can J Chem Eng.

[112] Wu HS, Wang YJ (2012). Salting-out effect on recovery of 1,3-propanediol from fermentation broth. Ind Eng Chem Res.

[113] Hongxin F, Yaqin S, Zhilong X (2013). Continuous countercurrent salting-out extraction of 1,3-propanediol from fermentation broth in a packed column. Process Biochem.

[114] Günzel B, Berke CH, Ernst S, Weitkamp J, Deckwer WD (1990). Adsorption von Diolen aus Fermentationsmedien an hydrophobe Zeolithe. Chem Ing Tech.

[115] Schöllner R, Einicke WD, Unverricht S, Brettner E (1994). Investigations of adsorptive separation of glycerol/propane-1,3-diol in aqueous solution on zeolites by liquid phase adsorption. J Prakt Chem.

[116] Anvari M, Khayati G (2009). In situ recovery of 2,3-butanediol from fermentation by liquid–liquid extraction. J Ind Microbiol Biotechnol.

[117] Pahlavanzadeh H, Khayati G, Ghaemi Nasser VFE (2012). Liquid-liquid extraction of 2,3-butanediol from fermentation broth. Iran J Chem Chem Eng.

[118] Greve A, Kula MR (1991). Cost structure and estimation for the recycling of salt in a protein extraction process. Bioprocess Eng.

[119] Tianwei T, Qing H, Qiang L (2002). Purification of glycyrrhizin from Glycyrrhiza uralensis Fisch with ethanol/phosphate aqueous two phase system. Biotechnol Lett.

[120] Zhi W, Deng Q (2006). Purification of salvianolic acid B from the crude extract of Salvia miltiorrhiza with hydrophilic organic/salt-containing aqueous two-phase system by counter-current chromatography. J Chromatogr A.

[121] Li Z, Jiang B, Zhang D, Xiu Z (2009). Aqueous two-phase extraction of 1,3-propanediol from glycerol-based fermentation broths. Sep Purif Technol.

[122] Li Z, Teng H, Xiu Z (2011). Extraction of 1,3-propanediol from glycerol-based fermentation broths with methanol/phosphate aqueous two-phase system. Process Biochem.

[123] Aydoğan Ö, Bayraktar E, Mehmetoğlu Ü, Kaeding T, Zeng AP (2010). Selection and optimization of an aqueous two-phase system for the recovery of 1,3-propandiol from fermentation broth. Eng Life Sci.

[124] Müller A, Górak A (2012). Extraction of 1,3-propanediol from aqueous solutions using different ionic liquid-based aqueous two-phase systems. Sep Purif Technol.

[125] Müller A, Schulz R, Wittmann J, Kaplanow I, Górak A (2013). Investigation of a phosphate/1-butyl-3-methylimidazolium trifluoromethanesulfonate/water system for the extraction of 1,3-propanediol from fermentation broth. RSC Adv.

[126] Müller A, Lutze P, Górak A (2013). Experimental and theoretical investigation of multistage extraction of 1,3-propanediol using the extraction system phosphate/1-butyl-3-methylimidazolium trifluoromethanesulfonate/water. Biotechnol Prog.

[127] Ghosh S, Swaminathan T (2003). Optimization of process variables for the extractive fermentation of 2,3-butanediol by *Klebsiella oxytoca*in aqueous two-phase system using response surface methodology. Chem Biochem Eng Q.

[128] Ghosh S, Swaminathan T (2004). Optimization of the phase system composition of aqueous two-phase system for extraction of 2,3-butanediol by theoretical formulation and using response surface methodology. Chem Biochem Eng Q.

[129] Sun LH, Jiang B, Xiu ZL (2009). Aqueous two-phase extraction of 2,3-butanediol from fermentation broths by isopropanol/ammonium sulfate system. Biotechnol Lett.

[130] Jiang B, Li ZG, Dai JY, Zhang DJ, Xiu ZL (2009). Aqueous two-phase extraction of 2,3-butanediol from fermentation broths using an ethanol/phosphate system. Process Biochem.

[131] Li Z, Teng H, Xiu Z (2010). Aqueous two-phase extraction of 2,3-butanediol from fermentation broths using an ethanol/ammonium sulfate system. Process Biochem.

[132] Anand P, Saxena RK, Marwah RG (2011). A novel downstream process for 1,3-propanediol from glycerol-based fermentation. Appl Microbiol Biotechnol.

[133] Barski P, Kowalczyk J, Lindstaedt A, Puzewicz-Barska J, Witt D (2012). Evaluation of solid phase extraction for downstream separation of propane-1,3-diol and butan-1-ol from fermentation broth. Process Biochem.

[134] Shao P, Kumar A (2009). Recovery of 2,3-butanediol from water by a solvent extraction and pervaporation separation scheme. J Membr Sci.

[135] Shao P, Kumar A (2009). Separation of 1-butanol/2,3-butanediol using ZSM-5 zeolite-filled polydimethylsiloxane membranes. J Membr Sci.

[136] Shao P, Kumar A (2011). Process energy efficiency in pervaporative and vacuum membrane distillation separation of 2,3-butanediol. Can J Chem Eng.

[137] Simpson SD, Fleming SE, Havill AM, RTrevethick S (2012). Process for producing chemicals using microbial fermentation of substrates comprising carbon monoxide.

[138] Li Y, Wu Y, Zhu J, Liu J, Shen Y: Separating 2,3-Butanediol from Fermentation Broth using n-Butylaldehyde. *J Saudi Chem Soc* 2013, http://dx.doi.org/10.1016/j.jscs.2013.02.005.,

[139] Clark W, Japs M, Burk M (2010). Process of Separating Components of a Fermentation Broth.

