# Supplementary figures and images for: Microbial production of short chain diols
Source: Microb Cell Fact. 2014 Dec 10;13:165. doi: 10.1186/s12934-014-0165-5 (PMC4269916; doi:10.1186/s12934-014-0165-5)

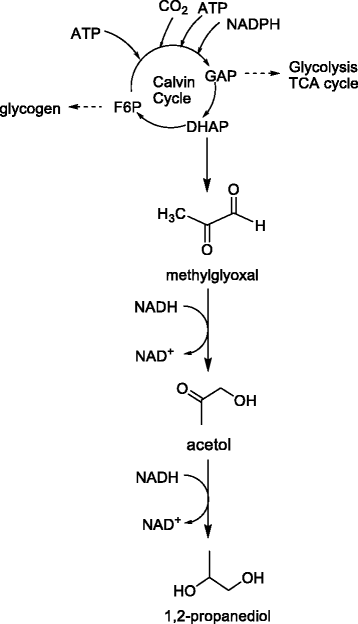

Supplement: Supplementary file 1 — Authors’ original file for figure 1 [file 12934_2014_165_MOESM1_ESM.gif]

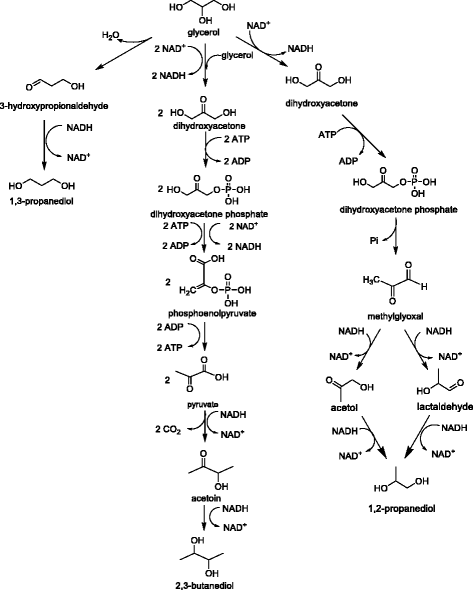

Supplement: Supplementary file 2 — Authors’ original file for figure 2 [file 12934_2014_165_MOESM2_ESM.gif]

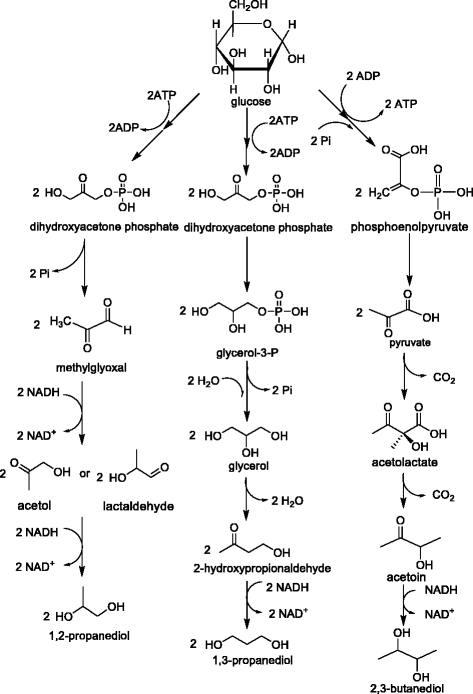

Supplement: Supplementary file 3 — Authors’ original file for figure 3 [file 12934_2014_165_MOESM3_ESM.gif]

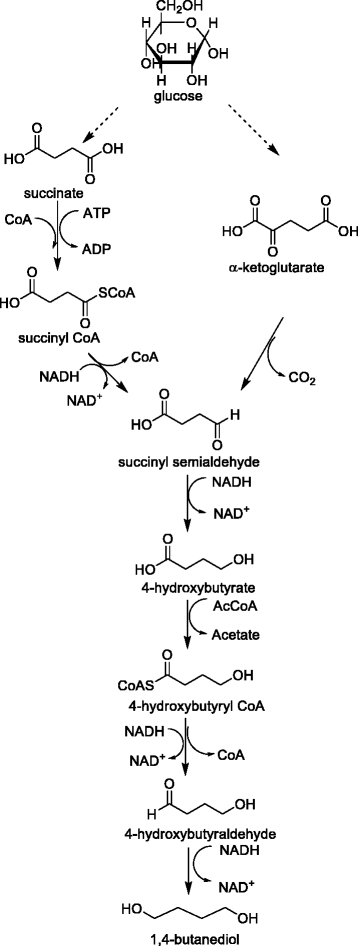

Supplement: Supplementary file 4 — Authors’ original file for figure 4 [file 12934_2014_165_MOESM4_ESM.gif]

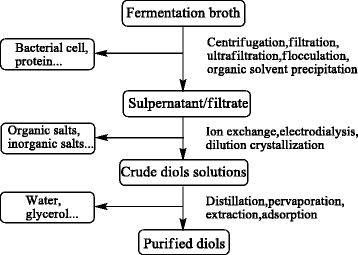

Supplement: Supplementary file 5 — Authors’ original file for figure 5 [file 12934_2014_165_MOESM5_ESM.gif]
